# 

**Published:** 1912-08-03

**Authors:** 


### The London School of Tropical Medicine.

The following have passed with distinction the examination of the 39th session, May-July, at the London School of Tropical Medicine :—Major W. P. Chamberlain (U.S. Army), M.D. (U.S.A.); Captain W. Lapsley (I.M.S.), M.B., R.U.I.; F. C. McCombie, M.D. (Lond.); Captain H. Emslie Smith (I.M.S.), M.B., Ch.B. (Aber.); Major H. R. Price (I.M.S.), M.B., F.R.C.S.E.; W. Allen (Colonial Service), M.B., Ch.B., D.P.H.; S. L. Brohier (Colonial Service), M.R.C.S., L.R.C.P., D.P.H.; W. J. Geale, L.R.C.P. and S. (Edin.); J. E. L. Johnston (Colonial Service), M.B., B.S. (Lond.), M.R.C.S., L.R.C.P.; A. H. Owen (Colonial Service), M.R.C.S., L.R.C.P.; B. Spearman (Colonial Service), M.B., B.C. (Camb.).

### The Indian Medical Service.

### THE SUCCESSFUL CANDIDATES FOR COMMISSIONS.

The following are the successful candidates in the competition for commissions in the Indian Medical Service, which was held at the Royal Army Medical College, and at the Examination Hall, Victoria Embankment, last week :—

J. D. Wilson, M.A., M.B., Ch.B.Edin.; L. A. P. Anderson, B.A., B.C., Cambridge University and St. George's Hospital; W. C. Paton, M.A., M.B., Ch.B. Edin.; J. B. Hance, B.A., M.B., B.C., M.R.C.S., L.R.C.P., Cambridge University and Guy's Hospital; S. Gordon, B.A., B.C., M.R.C.S., L.R.C.P., Cambridge University and London Hospital; G. Y. Thomson, M.B., B.S.Lond., M.R.C.S., L.R.C.P., Guy's Hospital; H. K. Rowntree, M.B., B.S.Lond., L.M.S.S.A., Middlesex Hospital; B. F. Eminson, M.B., B.S.Lond., Charing Cross Hospital; A. Kennedy, B.A., B.C., M.R.C.S., L.R.C.P., Cambridge University and Middlesex Hospital; J. C. John, B.A., M.B., B.C., M.R.C.S., L.R.C.P., Cambridge University and St. Bartholomew's Hospital; S. D. Ratnagar, B.A., L.M. and S., L.R.C.P. and S. Edin., L.F.P. and S. Glasg., London Hospital; C McIver, M.R.C.S., L.R.C.P., University College Hospital.

### Appointments.

Mr. F. A. Burke, Dr. Steevens' Hospital, Dublin, has been appointed assistant house surgeon at the District Hospital, West Bromwich.

Mr. Sidney Arthur Boyd, M.S. (Lond.), F.R.C.S. (Eng.), has been appointed surgeon to out-patients at the Hampstead General and North-West London Hospital.

The following appointments have been made at the University of Sheffield: Mr. Harold Leader, M.B., M.R.C.S., L.R.C.P., to the post of lecturer in diseases of children; Mr. H. Nield, M.B., Ch.B., to the post of demonstrator in anatomy; and Mr. E. F. Finch, M.D., F.R.C.S., and Mr. P. A. Reckless, F.R.C.S., to be honorary demonstrators in anatomy.

Mr. Donald Armour, F.R.C.S., has been appointed full surgeon at the West London Hospital. Mr. Oswald L. Addison, F.R.C.S., and Mr. Harry Tyrrell Gray, F.R.C.S., have been appointed assistant surgeons, and Mr. Sydney Gray MacDonald, F.R.C.S., surgical registrar. Dr. Seymour Taylor has been appointed consulting physician and Mr. F. Swinford Edwards, F.R.C.S., consulting surgeon to the hospital.

The following officers have been reappointed at Manchester Royal Infirmary: Mr. Edward Moir, L.S.A. (Lond.), and Mr. E. Falkner Hill, M.B., Ch.B. (Vict.), anæsthetists; Mr. A. E. Woodall, M.D., medical officer to the central branch; Mr. J. P. Buckley, F.R.C.S. (Eng.), and Mr. W. R. Douglas, F.R.C.S. (Eng.), assistant surgical officers; and Mr. F. H. Westmacott, F.R.C.S. (Eng.), assistant surgical officer, aural department.

### Books Received.

T. C. and E. C. Jack: "Cookery for Invalids," by Florence B. Jack.

John Bale, Sons and Danielsson: "Medical Gymnastics and Massage," by F. F. Middleweek, L.R.C.P.

Smith, Elder and Co.: "A Nurse's Life in War and Peace," by Miss E. C. Laurence.

### BUREAU OF INFORMATION.

### Rules for Correspondents.

1. Every letter, on whatever subject, must be accompanied by the coupon to be cut from the back cover (inside page) of The Hospital, current issue, and must contain the name and address of the correspondent with pseudonym for publication if desired.
2. Replies by post cannot be given, save under exceptional circumstances at the Editor's discretion.
3. Letters from Approved Homes in reply to special needs published in the Bureau should state terms and full particulars, and be sent prepaid under cover to the Editor of the Bureau with coupon and name enclosed for identification.
4. Proprietors of homes which have not yet been entered on the List of Approved Homes, but have spare accommodation likely to suit special needs, are invited to write for an application form for registration. The fee for registration is 5s.
5. Special needs of every description relating to those who are sick or in difficulties are made known in these columns free of charge.
6. All communications to be addressed to The Editor of The Hospital, 28 Southampton Street, Strand, London, W.C., and marked "Bureau of Information."

### Approved Homes.

Note.—*No Home is added to the List without direct medical and other testimony to its good management.*

### Nursing Home in Chesterfield.

120 Old Hall Road, Chesterfield.—Proprietor, Sister "Pam," trained nurse. Good house, standing in own grounds, free from noise. Medical, surgical, and nerve cases received. Terms very moderate.

### For Convalescents, etc., at New Milton.

Ashcombe House, New Milton, Hants.—Proprietor, Mrs. Brockell, trained nurse. Medical, surgical, and nerve cases received. Very invigorating air, and mild climate in winter. Good situation. Low terms to permanent patient.

### Private Home in Kent.

Mrs. Norman, Chelsfield, Kent, can receive lady or gentleman, at moderate terms, requiring quiet home and freedom from worry. Very suitable for after-care. Pretty country and good air.

### Books of Reference.

### On Preparation of Ligatures.

Sister M.—We advise you to procure either "Surgical Instruments and Appliances," price 1s. 6d., or "Surgical Ward Work and Nursing," price 5s., both published by The Scientific Press, 28 Southampton Street, Strand.

### Advanced Work on Bacteriology.

Post-Graduate.—The best German work to refer to is that by Kolle and Wassermann, "Handbuch der Pathogenischen Mikroorganismen" (Jena, 1904), with supplements still appearing from time to time. Consult also Muir and Ritchie's "Manual of Bacteriology," with Bibliography (Fourth Edition, 1908). On Immunity the best authorities are Ehrlich and Metchnikoff, and both of these may now be had in translation if preferred; the former, "Studies in Immunity," translated in New York, 1906; the latter, "Immunity in Infective Diseases," translated at Cambridge, 1905.

### Forms of Treatment.

### Sun-Baths.

Nurse B.—On no account allow your patient to attempt sun-baths without direct medical advice. For obvious reasons this treatment is less in vogue in this country than on the Continent, but with patience and under the doctor's orders it should not be impossible to carry through if desired. It may have very unfortunate results if improperly applied, and expose the patient to considerable local discomfort and even danger.

### Training and Employment.

### Secretarial Post.

B. B.—An opening occurs for well-educated lady secretary, to live in lodgings part of the year in London and part in the country. Good typing and shorthand. Knowledge of the procedure at committees desirable. Good letter-writing indispensable. Replies to B. B. under cover of the Bureau.

### Dentistry for Women.

Ignota.—To obtain the diploma of the Royal College of Surgeons in dental surgery a course of four years is necessary. The first two years can be carried out at the London School of Medicine for Women in connection with the Royal Free Hospital, Gray's Inn Road, London. The dental studies must then be undertaken for two years at a dental hospital, and at the National Dental Hospital, Great Portland Street, London, women students are received. The prospects for women dentists in this country are improving, and there are openings for work in female institutions. There are better openings, however, in India.

### Homes Wanted.

### For Chronic Invalid.

Stael (115a).—Accommodation required in nursing home by lady subject to occasional attacks of illness. Terms from £1 11s. 6d. to £2 2s. London preferred. Prepaid replies forwarded.

### For Patient with Loss of Memory.

M. E. I., Linthorpe.—You are quite right to make some fresh arrangement for your parent. It is not right you should be left entirely alone in the house with a patient who suffers from loss of memory, and your decision to earn your own living is, in our judgment, a wise one. We are making inquiries for you about a home, but fear it will not be easy to find. As you can pay 10s. a week, and the patient appears to give no trouble, you might find a reliable cottage home, but a good asylum would be safest and best in her interests. Consult your doctor about the fact of her being eligible for admission.

### Needs and Helpers.

Medical men needing accommodation at moderate terms for patients may be glad to know that we insert in these columns, free of charge, particulars of the requirements of those unable to pay full fees, and have a wide circle of homes willing to make reductions for patients of small means.

### Letter-Box.

S. M. (112a).—We understand sufficient answers have now been received to enable a choice to be made, and that no more are required. Cordial thanks are due to those whose generous offers cannot be accepted.

Nurse L. J.—Will reply fully next week.

M. E. I. B.—The nurse in question is now accommodated, but it is possible we may hear of some one to suit you. What payment do you require? Please supply reference.

"Grace."—We fear there is no remedy but patience and self-control.

**The leaflet containing particulars of the Bureau is now ready and will be forwarded free on application.**

